# Temporal trends of psychiatric disorders incidence by sex, education and immigration status among young and middle-aged adults in Sweden, 2004–2019

**DOI:** 10.1186/s12888-025-06596-8

**Published:** 2025-02-25

**Authors:** Ali Kiadaliri, Mehdi Osooli, Henrik Ohlsson, Jan Sundquist, Kristina Sundquist

**Affiliations:** 1 Department of Clinical Sciences Lund, Orthopaedics, Lund University, Skåne University Hospital, Remissgatan 4, Lund, SE-221 85 Sweden; 2 Division of Insurance Medicine, Department of Clinical Neuroscience, Karolinska Institute, Stockholm, Sweden; 3 Division of Clinical Epidemiology, Department of Medicine Solna, Karolinska Institute, Stockholm, Sweden; 4 Center for Primary Health Care Research, Department of Clinical Sciences, Lund University, Malmö, Sweden; 5 University Clinic Primary Care Skåne, Region Skåne, Malmö and Lund, Sweden

**Keywords:** Incidence, Intersectionality, Inequality, Psychiatric disorders, Temporal trend, Sweden

## Abstract

**Objective:**

To explore temporal changes in incidence of major psychiatric disorders across sociodemographic subgroups in Sweden.

**Methods:**

This population-based open cohort study included all individuals born during 1958–1994 and residing in Sweden at any time during 2004–2019. We identified psychiatric disorders registered in inpatient and outpatient specialist care. We calculated person-years from the inclusion until diagnosis of psychiatric disorder of interest, death, emigration or December 31, 2019, whichever occurred first. Combining sex (female, male), education (low, medium, high) and immigration status (first- or second-generation immigrant, native), we created a variable with 18 strata. Average annual percent changes (AAPCs) in age-standardized incidence rates (ASIRs) were estimated using Joinpoint regression.

**Results:**

A total of 5,051,875 individuals aged 25–61 years were followed for ≈ 56–58 million person-years. First-generation immigrants generally had lower overall ASIRs than second-generation and natives with more pronounced differences among persons with low education and females. While ASIRs of autism spectrum and other pervasive developmental disorders (AAPC 11.8%, 95% CI: 9.5, 15.8), as well as attention deficit hyperactivity disorder and conduct disorders (18.8%, 16.6, 25.0) rose over time, other psychiatric disorders were stable or had decreasing temporal changes (AAPC ranged from 0% for substance use disorders to -5.7% for schizophrenia/acute and transient psychotic disorders). First-generation immigrants generally experienced more favourable changes (i.e. more decreases or less increases) in ASIRs and this was most evident among those with low education.

**Conclusions:**

While incidence of psychiatric disorders in inpatient and outpatient specialist care generally declined during 2004–2019, there were important sociodemographic variations in temporal changes.

**Clinical trial number:**

Not applicable.

**Supplementary Information:**

The online version contains supplementary material available at 10.1186/s12888-025-06596-8.

## Introduction

Psychiatric disorders are one of the leading causes of disability worldwide [1]. Globally, 14.6% of the years lived with disability and 4.9% of the disability-adjusted life years were attributable to these disorders in 2019 [1]. The corresponding figures for Sweden were 16.1% and 7.9%, respectively [1]. Psychiatric disorders accounted for 53% and 42% of all sick leave cases among females and males, respectively, in Sweden in 2019 [2]. A recent study reported rising age- and sex-standardized prevalence of psychiatric disorders in the Stockholm Region, Sweden, during 2007–2017 [3]. Globally, while the crude burden of psychiatric disorders has increased over recent decades, the age-standardized burden has generally been stable/decreasing [4–6].

Inequalities in incidence and prevalence of psychiatric disorders by sociodemographic factors such as age, sex, race, socioeconomic status, migration, and place of residence are well-studied [7–11]. A few studies have also explored sociodemographic variations in temporal changes in incidence and prevalence of these disorders [11–14]. However, these studies focused either on a single sociodemographic factor or explored several sociodemographic factors independently, overlooking the fact that sociodemographic factors are interdependent, mutually constitutive, and reinforce one another [15]. Therefore, there is a lack of research on potential sociodemographic inequalities in temporal trends of psychiatric disorders at intersections of multiple sociodemographic factors. This study aims to address this knowledge gap by exploring the temporal changes in the incidence of major psychiatric disorders at intersections of sex, education, and immigration status using high-quality longitudinal population-based register data from Sweden.

## Methods

### Data sources

This was an observational population register-based open cohort study in Sweden. We obtained pseudonymized individual-level register data on birthyear, sex, date of death, highest educational attainment, and country of birth from Statistics Sweden. We retrieved data on all outpatient specialist and inpatient care from 2001 to 2019 from the National Patient Register (NPR) administered by the National Board of Health and Welfare [16]. The NPR contains the diagnostic codes according to the International Classification of Diseases 10 (ICD-10) system assigned by healthcare professionals at the time of the healthcare consultation. Data on familial linkage were obtained from the Multi-Generation Register [17]. Residents in Sweden receive a unique personal identity number (PIN) at birth or the time of immigration which was used to link their data across registers and over time. Statistics Sweden replaced all PINs with unique study numbers before delivering the data for this research.

### Study population

The study population included all individuals born between January 1, 1958, and December 31, 1994, and resided in Sweden at any point between January 1, 2004 and December 31, 2019. While we have data on the individuals born after the year 1994, we only included individual aged 25 + years to minimize the risk of misclassification for educational attainment. This means that the study sample covers the ages 25–61 years. We also excluded those with a previous diagnosis of the psychiatric disorder under investigation during 2001–2003. This was applied to mitigate the possibility of including prevalent cases. Moreover, individuals with missing information on own or parents’ country of birth and immigrants with missing migration date were excluded.

### Outcomes and follow-up

We identified persons with a first psychiatric disorder diagnosis registered in the NPR as a principal diagnosis. The following psychiatric disorders were separately investigated: substance use disorders (ICD-10 codes: F10–F19), schizophrenia and acute and transient psychotic disorders (ICD-10 codes: F20, F23), depressive disorders (ICD-10 codes: F32-F33), anxiety disorders (ICD-10 codes: F40-F41, F43), personality disorders (ICD-10 codes: F60, F63, F68), autism spectrum and other pervasive developmental disorders (ICD-10 code: F84), and attention deficit hyperactivity disorder (ADHD) and conduct disorders (ICD-10 codes: F90, F91). Each individual was followed from age 25 years, the date of immigration to Sweden or January 1, 2004 (whichever occurred last) until the first registration of the psychiatric disorder under investigation in the NPR, death, emigration from Sweden or December 31, 2019 (whichever occurred first).

### Sociodemographic strata

We included sex (female, male), education attainment (low: <10 years, medium: 10–12 years, and high: 13 + years) and immigration background (first-generation immigrants, second-generation immigrants, and native) as sociodemographic factors. First-generation immigrants were defined as those born abroad, the second-generation immigrants were individuals born in Sweden with at least one parent born abroad, and native were born in Sweden to two parents born in Sweden. Educational attainment was measured at the year of inclusion in the study. Using the possible unique combinations of these factors, we created 18 sociodemographic strata (18 = 2 × 3 × 3).

### Data analysis

For each sociodemographic stratum, we calculated the annual incidence rates as the number of new cases of the psychiatric disorder under investigation in each calendar year divided by the number of person-years of observation in that year. We also computed an overall incidence rate as the number of new cases of the psychiatric disorder under investigation during 2004–2019 divided by the total number of person-years during this period. We then estimated age-standardized incidence rates (ASIRs) using single age groups (25, 26, 27,…, 45, 46+) by means of direct standardization using the population of Sweden in the year 2010 as standard.

We used joinpoint regression to investigate temporal trends in ASIRs for each sociodemographic stratum using the Joinpoint Regression Program version 5.0.2 (https://surveillance.cancer.gov/joinpoint/). Given 16 data points (2004–2019) in our study, a maximum of 2 joinpoints and 3 trend segments were allowed. We set the minimum number as 3 observations per segment. Joinpoint regression, also known as change point regression or segmented regression, is a statistical method to identify change points in time series data (i.e. time points where model parameters change) [18]. It assumes that data points can be divided into segments, each with its own linear trend. It begins with estimating a model with 0 joinpoints (i.e. a straight line) and then tests whether adding more joinpoints results in improvements in the model fit. The optimal number of joinpoints (0–2) was selected based on data-driven weighted Bayesian Information Criteria (BIC). After selecting the optimal number of joinpoints, an annual percentage change for each segment was estimated. The weighted average of these annual percentage changes was then computed as the average annual percent change (AAPC) to summarize the trend for the whole period. Data visualizations were implemented in Stata v.18.

### Sensitivity analyses

We conducted two sensitivity analyses to account for the possible changes in the population composition over time. First, since our cohort was 25–46 years at the year 2004 (i.e. the starting year of the follow up), we conducted an analysis only in this specific age group to keep the cohort comparable throughout the follow up. Second, we conducted a closed cohort study, that is only individuals 25–46 years old at the year 2004 were included and followed.

## Results

A total of 5,051,875 individuals aged 25–61 years were included (48.9% females) and were followed for ≈ 56–58 million person-years. During the follow up, 296,427 incident cases of anxiety disorders, followed by 195,136 incident cases of depressive disorders and 113,364 incident cases of substance use disorders were registered in the NPR among the study population (Table 1). The proportion of individuals with an incident psychiatric disorder during follow up ranged from 0.4% for pervasive developmental disorders to 5.9% for anxiety disorder, corresponding to overall incidence rates of 3.2 and 53.8 per 10,000 person-years, respectively. Anxiety disorders had the highest overall age-standardized incidence rates (ASIRs) across all sociodemographic strata (Table 2). Depressive disorders had the second highest ASIRs across all strata except in second-generation immigrant males with low/medium education and native males with low education where substance use disorders had the second highest ASIRs.

**Table 1:** Number of persons and psychiatric disorders incidence in each sociodemographic stratum during 2004–2019

Stratum	*N*	Substance use disorders	Schizophrenia and acute and transient psychotic disorders	Depressive disorders	Anxiety disorders	Personality disorders	Autism spectrum and other pervasive developmental disorders	ADHD and conduct disorders
First-generation immigrant males with low education	118,951	3958	982	3973	7152	737	264	936
First-generation immigrant males with medium education	224,845	6990	1482	7828	12,314	1248	411	1810
First-generation immigrant males with high education	306,614	3818	869	6471	10,149	686	308	1237
First-generation immigrant females with low education	113,320	1576	725	5511	9091	662	146	517
First-generation immigrant females with medium education	195,617	3305	947	10,516	16,088	1341	328	1385
First-generation immigrant females with high education	332,310	2512	952	11,358	18,274	1132	356	1390
Second-generation immigrant males with low education	38,418	3139	594	2204	3424	770	500	1695
Second-generation immigrant males with medium education	153,320	6815	1095	6089	8740	1319	895	3245
Second-generation immigrant males with high education	112,184	2183	384	3290	4552	527	466	1145
Second-generation immigrant females with low education	23,029	1467	283	2014	3090	704	292	1124
Second-generation immigrant females with medium education	123,150	3665	595	7422	11,298	1773	617	2637
Second-generation immigrant females with high education	142,260	1927	384	6679	10,452	1150	439	1648
Native male low education	160,736	10,100	1536	8342	12,534	2086	1877	6075
Native male medium education	842,293	25,978	3377	27,489	38,764	4588	3890	13,076
Native male high education	622,310	9059	1530	15,532	21,898	1906	2232	4685
Native female low education	87,938	4602	834	7030	10,881	2283	1116	3922
Native female medium education	645,536	14,285	2090	32,223	48,852	6402	2582	10,703
Native female high education	809,044	7985	1560	31,165	48,874	4203	1865	6564
Total	5,051,875	113,364	20,219	195,136	296,427	33,517	18,584	63,794

ADHD: Attention deficit hyperactivity disorder

**Table 2:** Overall age-standardized incidence rates (95% CI) of specific psychiatric disorders per 10,000 person years during 2004–2019 across sociodemographic strata defined by nativity, sex, and education

Sociodemographic stratum	Substance use disorders	Schizophrenia and acute and transient psychotic disorders	Depressive disorders	Anxiety disorders	Personality disorders	Autism spectrum and other pervasive developmental disorders	ADHD and conduct disorders
First-generation immigrant males with low education	35.6 (34.4, 36.8)	8.3 (7.8, 8.9)	36.8 (35.6, 38.0)	66.5 (64.9, 68.1)	6.2 (5.8, 6.7)	2.1 (1.9, 2.4)	7.8 (7.2, 8.3)
First-generation immigrant males with medium education	29.6 (28.9, 30.3)	5.8 (5.5, 6.1)	33.4 (32.6, 34.1)	52.7 (51.7, 53.7)	4.9 (4.6, 5.1)	1.6 (1.4, 1.8)	7.1 (6.7, 7.4)
First-generation immigrant males with high education	13.0 (12.6, 13.5)	2.7 (2.5, 2.9)	23.0 (22.4, 23.7)	35.7 (34.9, 36.4)	2.3 (2.1, 2.5)	1.0 (0.9, 1.1)	3.8 (3.6, 4.0)
First-generation immigrant females with low education	14.3 (13.6, 15.0)	6.4 (5.9, 6.9)	52.2 (50.8, 53.7)	88.2 (86.4, 90.1)	5.7 (5.3, 6.2)	1.3 (1.0, 1.5)	4.4 (4.0, 4.8)
First-generation immigrant females with medium education	15.4 (14.9, 16.0)	4.2 (3.9, 4.5)	50.5 (49.5, 51.5)	78.8 (77.5, 80.0)	5.8 (5.5, 6.1)	1.4 (1.3, 1.6)	6.0 (5.7, 6.4)
First-generation immigrant females with high education	8.0 (7.7, 8.4)	2.8 (2.6, 3.0)	36.1 (35.3, 36.8)	59.0 (58.0, 59.9)	3.2 (3.0, 3.4)	1.1 (0.9, 1.2)	4.0 (3.7, 4.2)
Second-generation immigrant males with low education	77.0 (74.2, 79.8)	12.5 (11.5, 13.5)	49.4 (47.3, 51.5)	79.3 (76.6, 82.1)	16.0 (14.8, 17.2)	10.1 (9.2, 11.0)	35.4 (33.7, 37.1)
Second-generation immigrant males with medium education	40.2 (39.2, 41.2)	5.8 (5.5, 6.2)	34.4 (33.5, 35.3)	50.1 (49.0, 51.2)	7.0 (6.6, 7.4)	4.6 (4.3, 4.9)	17.4 (16.8, 18.1)
Second-generation immigrant males with high education	18.0 (17.2, 18.9)	2.6 (2.4, 2.9)	25.3 (24.3, 26.2)	35.5 (34.4, 36.6)	3.8 (3.4, 4.1)	3.3 (3.0, 3.7)	8.1 (7.6, 8.6)
Second-generation immigrant females with low education	60.2 (57.0, 63.3)	10.4 (9.2, 11.7)	80.1 (76.5, 83.6)	133.0 (128.2, 137.7)	24.8 (23.0, 26.7)	9.8 (8.7, 11.0)	40.4 (38.0, 42.8)
Second-generation immigrant females with medium education	26.3 (25.4, 27.1)	4.0 (3.7, 4.4)	52.5 (51.2, 53.7)	83.0 (81.5, 84.6)	11.6 (11.0, 12.1)	4.0 (3.7, 4.3)	17.3 (16.7, 18.0)
Second-generation immigrant females with high education	12.8 (12.2, 13.4)	2.2 (2.0, 2.5)	40.8 (39.7, 41.9)	66.8 (65.4, 68.2)	6.2 (5.8, 6.6)	2.6 (2.3, 2.9)	9.6 (9.1, 10.1)
Native male low education	54.7 (53.6, 55.8)	7.5 (7.2, 7.9)	43.2 (42.2, 44.1)	67.0 (65.8, 68.2)	10.3 (9.9, 10.8)	9.3 (8.9, 9.7)	31.1 (30.3, 31.9)
Native male medium education	26.5 (26.2, 26.8)	3.2 (3.1, 3.3)	26.7 (26.4, 27.0)	38.0 (37.6, 38.4)	4.2 (4.1, 4.3)	3.5 (3.4, 3.6)	12.1 (11.8, 12.3)
Native male high education	12.9 (12.6, 13.2)	1.8 (1.7, 1.9)	20.3 (20.0, 20.7)	29.1 (28.7, 29.6)	2.4 (2.3, 2.5)	2.7 (2.6, 2.8)	5.7 (5.6, 5.9)
Native female low education	45.8 (44.5, 47.1)	7.6 (7.1, 8.2)	71.1 (69.4, 72.8)	117.1 (114.9, 119.3)	21.4 (20.5, 22.3)	10.2 (9.6, 10.8)	37.1 (36.0, 38.3)
Native female medium education	18.3 (18.0, 18.6)	2.6 (2.5, 2.7)	41.3 (40.8, 41.7)	64.3 (63.7, 64.9)	7.8 (7.6, 8.0)	3.1 (3.0, 3.2)	13.0 (12.8, 13.3)
Native female high education	8.7 (8.5, 8.9)	1.6 (1.5, 1.7)	31.0 (30.6, 31.3)	50.7 (50.2, 51.2)	3.7 (3.6, 3.9)	1.8 (1.7, 1.9)	6.2 (6.0, 6.4)

ADHD: Attention deficit hyperactivity disorder

### Substance use disorders

**Fig. 1:**
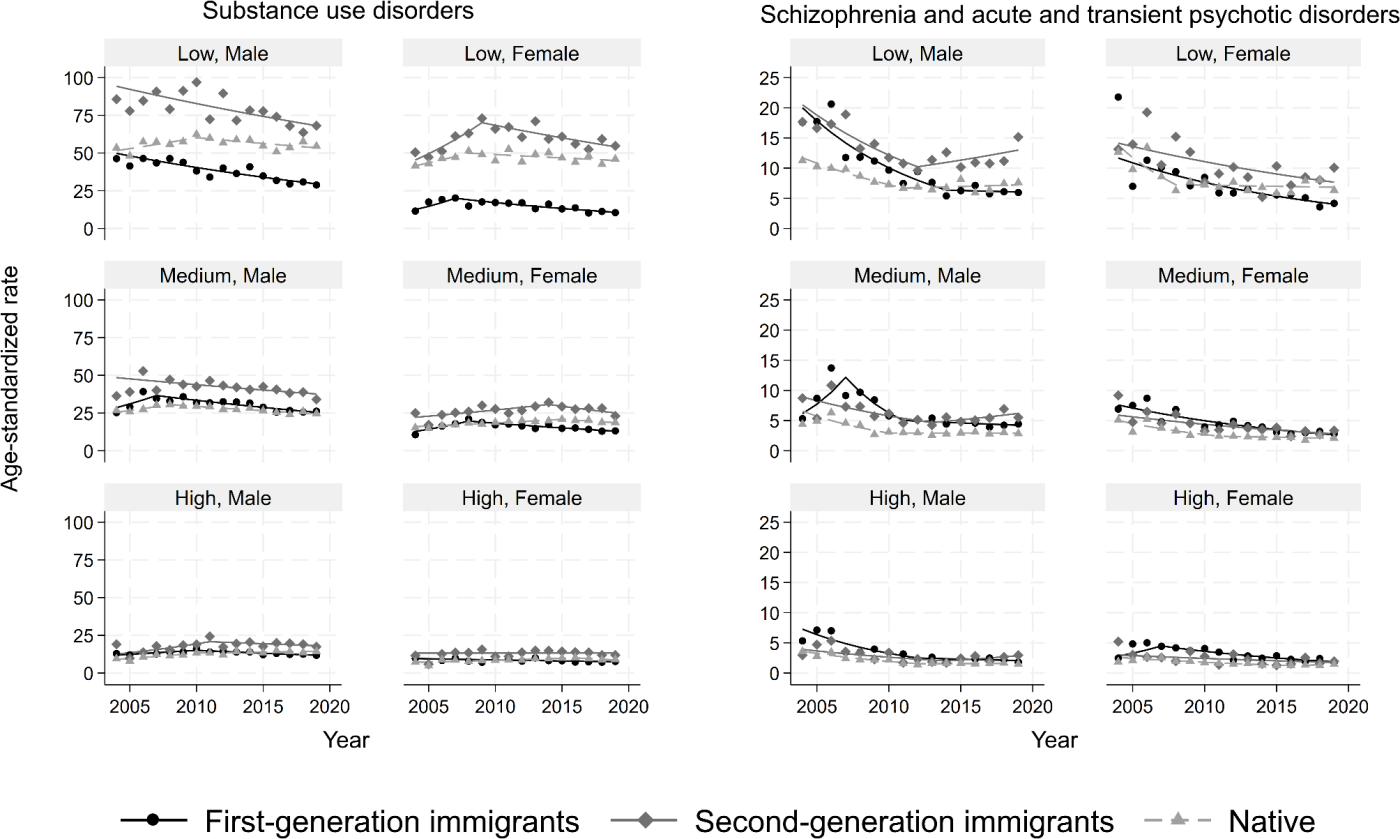
Age-standardized incidence rates of substance use disorders (left panel) and schizophrenia and acute and transient psychotic disorders (right panel) per 10,000 person-years, stratified by immigration, sex and education. Symbols display observed values and lines display the modelled values

The overall ASIRs per 10,000 person-years ranged from 8.0 (95% CI: 7.7, 8.4) in first-generation immigrant females with high education to 77.0 (74.2, 79.8) in second-generation immigrant males with low education (Table 2). The second-generation immigrants had the greatest ASIR regardless of sex and education, even though the differences became smaller as education increased (Fig. 1). On the other hand, first-generation immigrants had the lowest ASIR among those with low education, but the rates were comparable to natives among those with medium to high education. The average annual percent change (AAPC) ranged from a 3.4% (95% CI 2.1, 4.4) decline among first-generation immigrant males with low education to a 3.1% (95% CI 1.9, 5.7) rise for native males with high education (Table A1 in supplement).

### Schizophrenia and acute and transient psychotic disorders

The lowest and highest overall ASIRs per 10,000 person-years were observed in native females with high education (1.6, 95% CI 1.5, 1.7) and second-generation immigrant males with low education (12.5, 95% CI 11.5, 13.5), respectively. Natives generally had lower ASIRs than immigrants, even though their advantages disappeared among the low education subgroup in recent years (Fig. 1). The ASIRs declined in all strata over the whole study period with an AAPC of 2.0% in second-generation males with high education to 8.0% in first-generation males with high education (Table A2 in supplement). Several strata had rising trends in more recent years.

### Depressive disorders

**Fig. 2:**
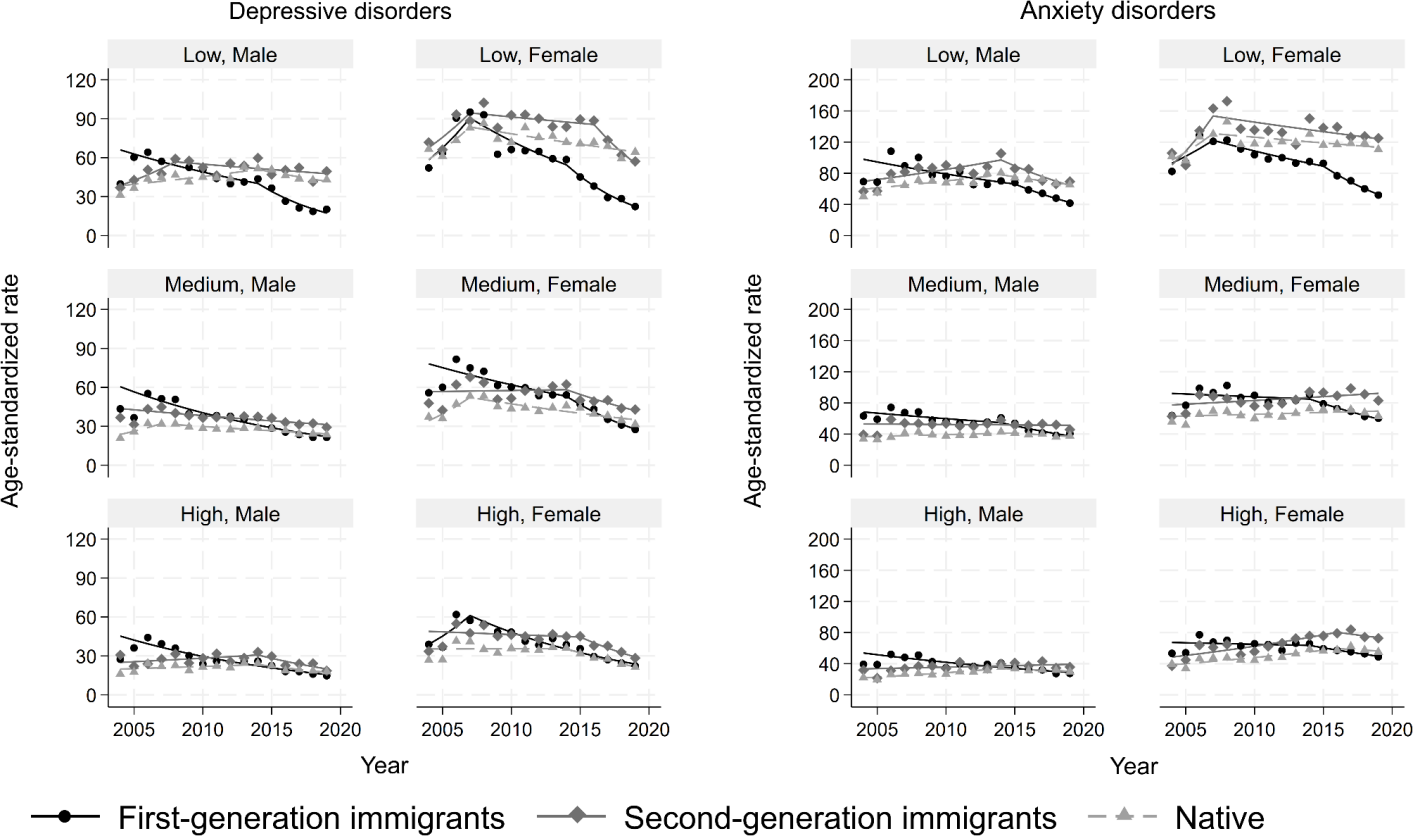
Age-standardized incidence rates of depressive (left panel) and anxiety (right panel) disorders per 10,000 person-years, stratified by immigration, sex and education. Symbols display observed values and lines display the modelled values

The overall ASIRs per 10,000 person-years ranged from 20.3 (95% CI: 20.0, 20.7) among native males with high education to 80.0 (76.5, 83.6) in second-generation immigrant females with low education. The ASIRs were generally declining with most and least reductions among first-generation immigrants and natives, respectively (Fig. 2 & Table A3 in supplement). While among those with medium and high education across both sexes, the immigrant-native gap narrowed over time, among persons with low education the gap widened in favour of first-generation immigrants.

### Anxiety disorders

Native males with high education and second-generation females with low education had the lowest (29.1, 95% CI: 28.7, 29.6) and highest (133.0, 95% CI: 128.2, 137.7) overall ASIRs, respectively. The ASIRs were declining among first-generation immigrants while were stable or rising among second-generation immigrants and natives (Fig. 2). Almost all first-generation immigrant subgroups experienced a joinpoint in their temporal trends in the years 2014–2015 (Table A4 in supplement). Persons with low education generally experienced more favourable changes than those with high education regardless of sex and immigration background.

### Personality disorders

**Fig. 3:**
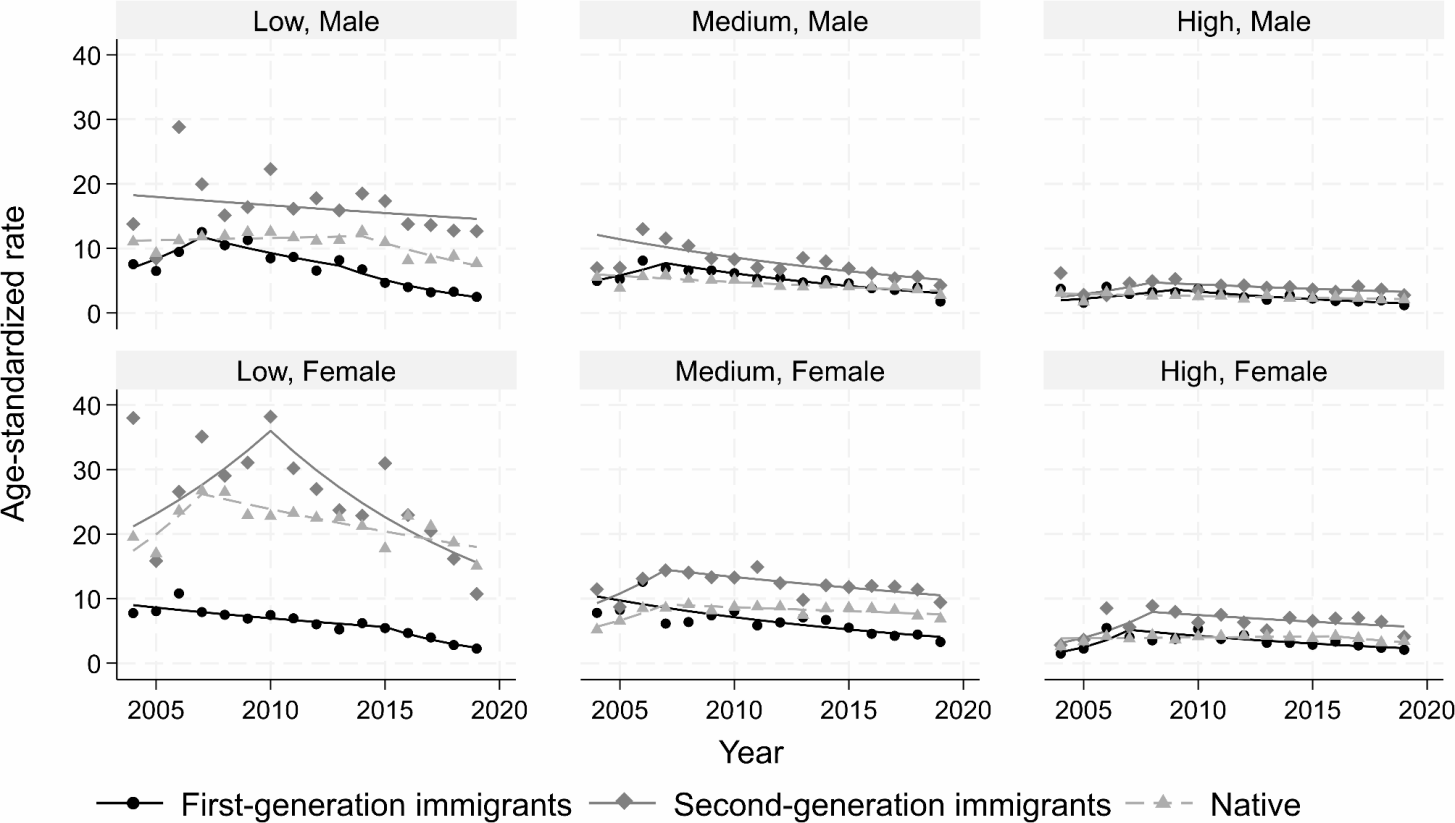
Age-standardized incidence rates of personality disorders per 10,000 person-years, stratified by immigration, sex and education. Symbols display observed values and lines display the modelled values

The overall ASIRs per 10,000 person-years ranged from 2.3 (95% CI: 2.1, 2.5) for first-generation immigrant males with high education to 24.8 (23.0, 26.7) for second-generation immigrant females with low education. The temporal trends were mostly declining or stable across sociodemographic strata (Table A5 in supplement). First-generation immigrants generally had the lowest ASIRs throughout the study period and this was more pronounced among people with low education (Fig. 3).

### Autism spectrum and other pervasive developmental disorders

**Fig. 4:**
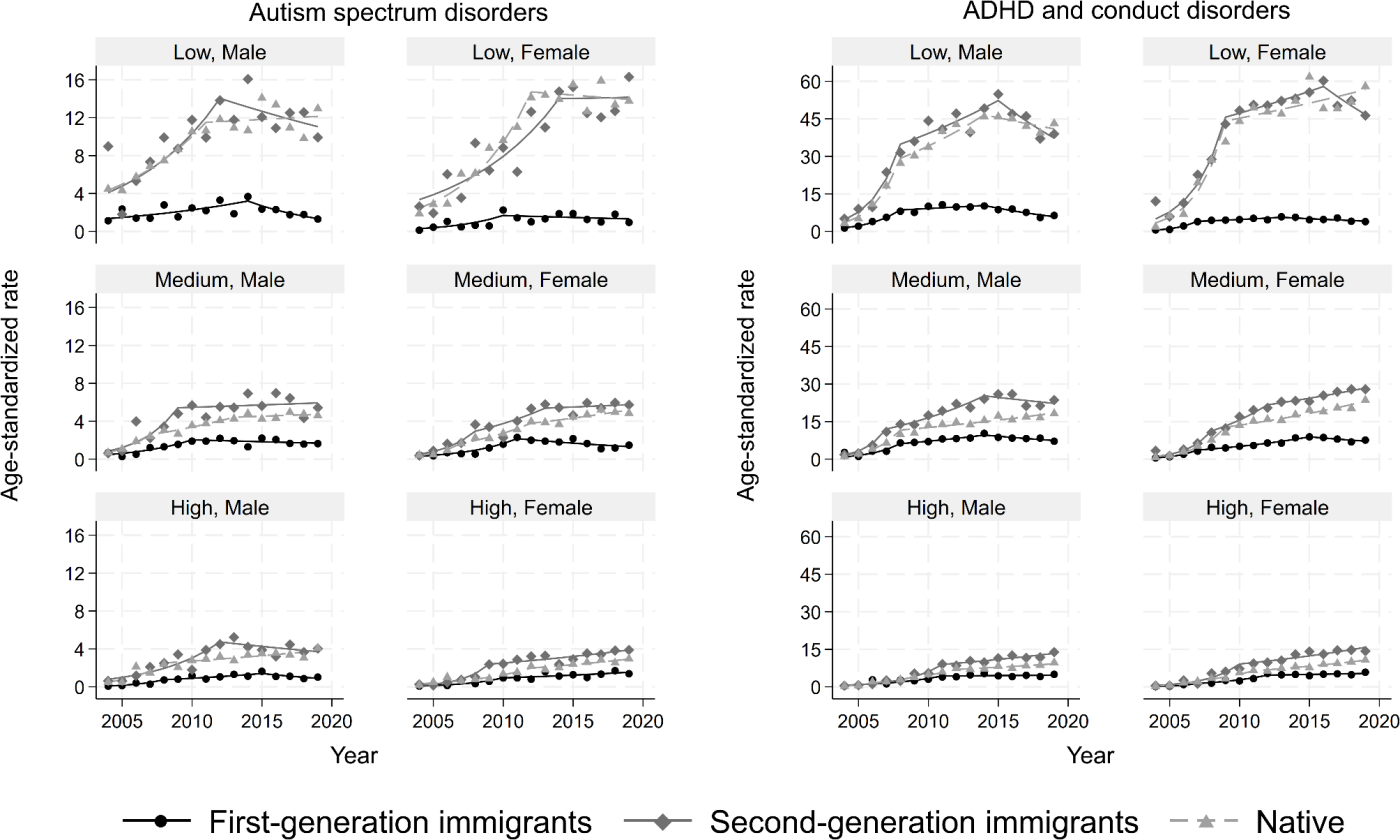
Age-standardized incidence rates of autism spectrum and other pervasive developmental disorder (left panel) and attention-deficit hyperactivity disorder and conduct disorder (right panel) per 10,000 person-years, stratified by immigration, sex and education. Symbols display observed values and lines display the modelled values

The overall ASIRs were higher among second-generation immigrants and natives with low education than in other sociodemographic strata. Almost all strata experienced rising trends in ASIRs over the study period (Fig. 4 & Table A6 in supplement). The first-generation immigrants had lower ASIRs than other strata and this gap was widening over time.

### ADHD and conduct disorders

The overall ASIRs per 10,000 person-years ranged from 3.8 (95% CI 3.6, 4.0) in first-generation immigrant males with high education to 40.4 (95% CI 38.0, 42.8) in second-generation females with low education. The ASIRs rose in all strata with more pronounced rises among second-generation immigrants and natives compared with first-generation immigrants (Fig. 4). Many strata experienced 3 joinpoints with declining trends for several strata in most recent years (Table A7 in supplement).

### Sensitivity analyses

The patterns of sociodemographic variations in temporal changes of psychiatric disorders incidence in our sensitivity analyses were generally similar to those observed in the main analysis (Tables A8–A11 in supplement).

## Discussion

This nationwide cohort study, for the first time, investigated potential inequalities in temporal trends of the incidence of psychiatric disorders diagnosed in outpatient specialist and inpatient care from an intersectionality perspective. For most studied psychiatric disorders, we observed stable or decreasing trends in age-standardized incidence rates, with important differences across sociodemographic strata defined by sex, education and immigration background. Specifically, first-generation immigrants generally experienced more favourable changes, and this was more pronounced among those with low educational attainment.

Previous research reported mixed findings on temporal trends of the incidence of psychiatric disorders [4–6, 13, 14, 19]. For example, two recent studies reported stable trends in crude incidence rates of registered common psychiatric disorders [14] and generalised anxiety and depression [20] in the UK, while increasing trends in crude and age-standardized rates of psychiatric disorders have been reported in Denmark [13, 19] over recent decades. On the other hand, several studies documented stable or decreasing trends in age-standardized incidence rates of psychiatric disorders, especially using the estimates from Global Burden of Disease Study [4, 5, 21, 22]. Differences in healthcare systems, underlying population, diagnosis coding practices (e.g. ICD version), time periods, methodology (e.g. age-standardization), data source (e.g. register, surveys), and level of care (i.e. primary vs. secondary care) might partially explain these mixed findings. It should also be noted that we only included treated psychiatric disorders while some of previous studies included both treated and untreated (e.g. measured using patient-reported outcomes) psychiatric disorders. These differences also highlight the difficulties in cross-study comparisons of incidence rates and their changes over time.

The observed changes in the incidence of diagnosed psychiatric disorders among young and middle-aged adults in the present study might reflect (i) true changes in the underlying mental health conditions, (ii) changes in the management of psychiatric symptoms at health care settings, including primary care (iii) changes in help-seeking behaviours, (iv) changes in medical technology and knowledge, (v) changes in stigmatisation and awareness, and/or (vi) changes in diagnostic criteria and coding practices [14]. Specifically, given that our data source didn’t cover primary care data, improvements in the management of psychiatric disorders at primary care and a shift toward delivery and receiving care at primary care settings would result in declining trends in receiving care at a secondary care setting. On the other hand, advances in diagnostic technology and greater medical knowledge can lead to better detection of psychiatric disorders that were previously neglected or missed (e.g. ADHD and autism).The increasing trends in ADHD and conduct disorders as well as autism in the adult population in the current study is in line with recent studies from Denmark [23], the UK [24] and the US [25]. These rising trends might be due to increased awareness, changes in recordings (e.g. lower symptom thresholds for a clinical diagnosis), and changes in perception of these disorders persisting beyond childhood and adolescence [23–25].

However, our results suggest important variations in temporal trends across sociodemographic strata which cannot be captured by exploring overall trends. Previous studies also documented variations in temporal trends of psychiatric disorders incidence rates by sociodemographic factors such as sex, age, and race [13, 14, 19, 20, 25]. For example, exploring age differences generally suggested more pronounced temporal changes in the incidence of psychiatric disorders among younger than older individuals [13, 14, 19, 20]. However, these studies treated sociodemographic factors independently which can mask important variations within a population. For instance, Chung et al. reported racial variations in temporal trends of ADHD incidence in the US which resulted in a narrowing racial difference between whites and other races over time [25]. In the present study, however, we observed that the changes in native-immigrant gaps over time varied across sexes and educational groups. For example, while native-immigrant gaps in the incidence of anxiety disorders narrowed among males with low education, it widened among females with low education. Therefore, our results provide a more detailed mapping of sociodemographic differences in the temporal trends of psychiatric disorders incidence compared with common unidimensional analyses focusing on a single sociodemographic factor. The observed sociodemographic disparities in temporal changes of diagnosed psychiatric disorders incidence might reflect sociodemographic differences in epidemiologic and clinical profile of these disorders, prevalence of psychiatric disorders’ risk factors including genetic predisposition, access to healthcare, and cultural differences which can influence health-seeking behaviours and coding practices [26].

Our results suggested that first-generation immigrants generally experienced more favourable changes (i.e. more decreases or less increases) in age-standardized incidence rates compared with second-generation immigrants and natives. Moreover, for most psychiatric disorders included in the present study, first-generation immigrants also had lower overall incidence rates during 2004–2019. However, these disparities in overall incidence and temporal changes were more pronounced among people with low level of education in both sexes. While not a universal phenomenon, several previous studies also reported lower risks of many psychiatric disorders among first-generation immigrants than second-generation immigrants and natives in Sweden and other countries [26–30]. We speculate that lower incidence rates and more favourable changes among first-generation immigrants might be partially explained by the “healthy immigrant effect” suggesting that foreign-born individuals tend to be healthier with better coping skills and higher resilience compared with the native-born population [29]. A recent study among individuals aged 18–47 years in Sweden documented a “healthy immigrant effect” for psychiatric disorders diagnosed in outpatient specialist and inpatient care among Western but not non-Western migrants [31]. Our findings might also reflect cultural and language barriers in the use of psychiatric care services among first-generation immigrants [26, 32]. This explanation is further supported by more pronounced differences among those with low educational attainment who might encounter more cultural, and language barriers compared with their counterparts with higher education. Moreover, increased availability and accessibility of digital care allowing receiving remote consultations from their home country might have also contributed to more favourable changes among first-generation immigrants. While unlikely, the possibility of a more pronounced shift toward the use of primary care among the first-generation immigrants compared with second-generation immigrants and natives cannot be fully ruled out given the lack of data on primary care in the present study. However, this cannot explain the observed differences for more severe psychiatric disorders such as psychosis and schizophrenia which are mainly managed in specialist care. Another possible explanation is the “salmon bias” hypothesis which states that certain immigrant population tend to return to their country of origin when they become seriously ill [33]. However, this hypothesis has been challenged in a recent study in Sweden, even though there was evidence of a partial “salmon bias” effect for immigrants from the Horn of Africa, the Rest of Africa, the Middle East and Eastern Europe and Russia [33]. These results may suggest a need of revising Sweden’s integration policies and call for increased intersectional awareness in public health policymaking. They can also be used to guide targeted mental health interventions towards the most vulnerable groups, especially second-generation immigrants with low educational attainment.

The use of nationwide high quality longitudinal register data with minimal recall and selection biases as well as employing an intersectional perspective to explore temporal changes are the main strength of the present study. However, our findings should be interpreted in light of several limitations. While the NPR is a valid and reliable data source [16], it is still prone to misdiagnosis and coding errors and possible sociodemographic variations in these errors can bias our estimates. The lack of data on primary care consultations implies that our results are an underestimate of the psychiatric disorders incidence in Sweden. We also lack data on incidence among those born prior to the year 1958 and hence the results are not generalizable to older cohorts. To avoid low statistical power, we did not consider the specific country of origin and time since immigration when identifying first- and second-generation immigrants. However, we expect variations in temporal changes within these subgroups which should be considered when interpreting the findings. A similar caution applies to age groups, that is there can be variations in temporal changes across age groups which is not captured by age-standardized rates. As this is a descriptive epidemiological study, all potential explanations of possible mechanisms are therefore speculative.

## Conclusion

This nationwide study suggested a generally stable or declining trends in age-standardized incidence rates of several psychiatric disorders diagnosed in outpatient specialist and inpatient care among young and middle aged adults in Sweden during 2004–2019. The study revealed important sociodemographic disparities in these temporal changes. Specifically, first-generation immigrants generally experienced more favourable changes than second-generation immigrants and natives with more pronounced disparities among those with low education. To provide a more comprehensive picture of psychiatric disorder incidence in the country, future studies could explore temporal changes in psychiatric disorders diagnosed in primary care settings.

## Electronic supplementary material

Below is the link to the electronic supplementary material.

Supplementary Material 1

## Data Availability

“According to the Swedish law, national register data cannot be shared, but it is possible to apply for their use from the authorities.”

## Abbreviations

AAPC: Average annual percent change
ADHD: Attention deficit hyperactivity disorder
ASIR: Age-standardized incidence rate
BIC: Bayesian information criteria
ICD: International classification of diseases
NPR: National patient register
PIN: Personal identity number
