# Supplementary material for: Temporal trends of psychiatric disorders incidence by sex, education and immigration status among young and middle-aged adults in Sweden, 2004–2019
Source: BMC Psychiatry. 2025 Feb 25;25:166. doi: 10.1186/s12888-025-06596-8 (PMC11863446; doi:10.1186/s12888-025-06596-8)
Supplement: Supplementary file 1 — Supplementary Material 1 [file 12888_2025_6596_MOESM1_ESM.pdf]

Table A1. Temporal changes in age-standardized rates of substance use disorders across sociodemographic strata defined by nativity, sex, and education, 2004-2019.

Stratum	Period 1	APC 1	Period 2	APC 2	AAPC (2004–2019)
First-generation immigrant males with low education	2004–2019	-3.4 (-4.4, -2.2)			-3.4 (-4.4, -2.2)
First-generation immigrant males with medium education	2004–2007	8.6 (-3.3, 24.7)	2007–2019	-3.0 (-6.9, -1.1)	-0.8 (-2.9, 1.9)
First-generation immigrant males with high education	2004–2010	3.9 (0.8, 14.7)	2010–2019	-2.7 (-4.1, -1.7)	-0.1 (-1.0, 1.9)
First-generation immigrant females with low education	2004–2007	17.0 (0.5, 36.6)	2007–2019	-5.1 (-9.0, -3.4)	-1.0 (-3.3, 1.9)
First-generation immigrant females with medium education	2004–2008	11.4 (3.5, 28.9)	2008–2019	-3.7 (-5.0, -2.7)	0.1 (-1.2, 2.4)
First-generation immigrant females with high education	2004–2019	-1.6 (-3.1, 0.6)			-1.6 (-3.1, 0.6)
Second-generation immigrant males with low education	2004–2019	-2.1 (-3.6, -0.5)			-2.1 (-3.6, -0.5)
Second-generation immigrant males with medium education	2004–2019	-1.7 (-2.5, -0.6)			-1.7 (-2.5, -0.6)
Second-generation immigrant males with high education	2004–2011	7.5 (2.9, 35.4)	2011–2019	-1.7 (-6.9, 0.6)	2.5 (0.5, 7.2)
Second-generation immigrant females with low education	2004–2009	9.1 (4.1, 21.0)	2009–2019	-2.5 (-4.5, -1.3)	1.2 (-0.0, 3.2)
Second-generation immigrant females with medium education	2004–2014	3.3 (1.8, 8.3)	2014–2019	-4.0 (-9.2, -0.8)	0.8 (-0.2, 2.6)
Second-generation immigrant females with high education	2004–2019	0.0 (-1.8, 2.8)			0.0 (-1.8, 2.8)
Native male low education	2004–2010	2.6 (0.4, 12.2)	2010–2019	-1.3 (-5.2, -0.3)	0.2 (-0.9, 1.8)
Native male medium education	2004–2008	4.8 (0.7, 13.1)	2008–2019	-2.0 (-2.8, -1.5)	-0.3 (-1.0, 0.9)
Native male high education	2004–2010	6.5 (3.1, 22.5)	2010–2019	0.9 (-2.0, 2.1)	3.1 (1.9, 5.7)
Native female low education	2004–2008	4.7 (0.1, 14.7)	2008–2019	-0.9 (-4.7, 0.0)	0.5 (-0.8, 2.1)
Native female medium education	2004–2015	2.4 (1.8, 3.9)	2015–2019	-2.8 (-6.2, -0.6)	1.0 (0.5, 1.8)
Native female high education	2004–2016	2.9 (2.0, 12.5)	2016–2019	-4.7 (-9.5, 0.7)	1.4 (0.5, 4.0)

APC: Annual percentage change; AAPC: Average annual percent change.

Numbers in parentheses show 95% confidence intervals.

Table A2. Temporal changes in age-standardized rates of schizophrenia and acute and transient psychotic disorders across sociodemographic strata defined by nativity, sex, and education, 2004-2019.

Stratum	Period 1	APC 1	Period 2	APC 2	Period 3	APC 3	AAPC (2004–2019)
First-generation immigrant males with low education	2004–2014	-10.8 (-19.4, -8.1)	2014–2019	-1.0 (-6.8, 9.4)			-7.7 (-9.9, -5.8)
First-generation immigrant males with medium education	2004–2007	25.5 (7.6, 44.5)	2007–2011	-19.8 (-25.4, -11.3)	2011–2019	-2.1 (-5.3, 7.5)	-2.5 (-4.4, 0.1)
First-generation immigrant males with high education	2004–2012	-12.5 (-27.1, -6.9)	2012–2019	-2.6 (-7.2, 8.8)			-8.0 (-10.7, -5.1)
First-generation immigrant females with low education	2004–2019	-6.8 (-8.6, -4.8)					-6.8 (-8.6, -4.8)
First-generation immigrant females with medium education	2004–2019	-6.8 (-9.2, -3.9)					-6.8 (-9.2, -3.9)
First-generation immigrant females with high education	2004–2007	16.9 (-2.5, 40.6)	2007–2019	-7.1 (-14.8, -4.7)			-2.7 (-5.8, 0.7)
Second-generation immigrant males with low education	2004–2012	-8.3 (-18.4, -4.0)	2012–2019	3.5 (-0.6, 13.9)			-3.0 (-5.2, -0.9)
Second-generation immigrant males with medium education	2004–2013	-6.9 (-19.3, -2.7)	2013–2019	4.9 (-0.9, 18.5)			-2.4 (-5.1, 0.1)
Second-generation immigrant males with high education	2004–2014	-6.4 (-23.9, 15.1)	2014–2019	7.5 (-5.4, 30.5)			-2.0 (-5.6, 2.2)
Second-generation immigrant females with low education	2004–2019	-4.0 (-6.8, -1.2)					-4.0 (-6.8, -1.2)
Second-generation immigrant females with medium education	2004–2019	-4.9 (-7.8, -1.7)					-4.9 (-7.8, -1.7)
Second-generation immigrant females with high education	2004–2019	-3.5 (-8.4, 2.4)					-3.5 (-8.4, 2.4)
Native male low education	2004–2011	-7.7 (-12.7, -4.6)	2011–2019	0.9 (-1.2, 5.3)			-3.2 (-4.4, -1.9)
Native male medium education	2004–2010	-12.5 (-23.5, -6.1)	2010–2019	-0.1 (-2.5, 5.9)			-5.3 (-7.2, -2.9)
Native male high education	2004–2012	-10.0 (-18.6, -6.3)	2012–2019	0.3 (-3.1, 8.3)			-5.3 (-7.2, -3.5)
Native female low education	2004–2008	-14.8 (-27.8, -1.7)	2008–2019	-0.5 (-6.8, 12.6)			-4.6 (-6.8, -1.1)
Native female medium education	2004–2011	-10.0 (-20.9, -5.5)	2011–2019	-1.8 (-4.8, 6.9)			-5.7 (-8.0, -3.6)
Native female high education	2004–2016	-6.0 (-12.7, 0.7)	2016–2019	4.9 (-4.7, 10.7)			-3.9 (-5.9, -2.4)

APC: Annual percentage change; AAPC: Average annual percent change.

Numbers in parentheses show 95% confidence intervals.

Table A3. Temporal changes in age-standardized rates of depressive disorders across sociodemographic strata defined by nativity, sex, and education, 2004-2019.

Stratum	Period 1	APC 1	Period 2	APC 2	Period 3	APC 3	AAPC (2004–2019)
First-generation immigrant males with low education	2004–2014	-4.9 (-7.0, 8.2)	2014–2019	-15.5 (-26.6, -10.1)			-8.5 (-10.7, -4.8)
First-generation immigrant males with medium education	2004–2019	-6.5 (-7.9, -4.9)					-6.5 (-7.9, -4.9)
First-generation immigrant males with high education	2004–2019	-6.8 (-8.2, -5.0)					-6.8 (-8.2, -5.0)
First-generation immigrant females with low education	2004–2007	15.8 (2.1, 33.4)	2007–2014	-7.0 (-10.0, -4.0)	2014–2019	-16.3 (-24.4, -13.0)	-6.2 (-8.3, -3.7)
First-generation immigrant females with medium education	2004–2014	-3.8 (-5.2, -0.6)	2014–2019	-12.2 (-17.6, -9.0)			-6.7 (-7.8, -5.3)
First-generation immigrant females with high education	2004–2007	16.2 (-4.8, 38.3)	2007–2019	-7.6 (-10.5, -6.3)			-3.2 (-6.6, 0.0)
Second-generation immigrant males with low education	2004–2008	10.2 (1.7, 26.4)	2008–2019	-1.6 (-4.7, -0.3)			1.4 (-0.4, 3.8)
Second-generation immigrant males with medium education	2004–2019	-2.2 (-3.1, -1.1)					-2.2 (-3.1, -1.1)
Second-generation immigrant males with high education	2004–2014	2.1 (-0.2, 28.1)	2014–2019	-8.2 (-19.1, -2.9)			-1.5 (-3.5, 4.4)
Second-generation immigrant females with low education	2004–2007	11.8 (2.2, 23.1)	2007–2016	-1.1 (-2.7, 0.5)	2016–2019	-13.4 (-17.9, -7.8)	-1.3 (-2.9, 0.6)
Second-generation immigrant females with medium education	2004–2014	0.3 (-2.4, 27.6)	2014–2019	-6.3 (-18.1, -1.3)			-2.0 (-4.1, 2.9)
Second-generation immigrant females with high education	2004–2015	-0.8 (-2.0, 2.1)	2015–2019	-10.1 (-15.7, -5.9)			-3.4 (-4.4, -1.9)
Native male low education	2004–2014	2.9 (1.5, 14.5)	2014–2019	-3.9 (-12.7, -0.1)			0.6 (-1.0, 3.2)
Native male medium education	2004–2007	10.6 (-0.7, 25.1)	2007–2019	-2.3 (-5.1, -1.4)			0.1 (-1.9, 2.5)
Native male high education	2004–2014	1.6 (0.0, 16.3)	2014–2019	-5.7 (-13.3, -2.0)			-0.9 (-2.3, 2.6)
Native female low education	2004–2007	12.6 (1.9, 23.8)	2007–2019	-2.1 (-4.0, -1.3)			0.6 (-0.9, 2.4)
Native female medium education	2004–2007	15.1 (-0.4, 34.6)	2007–2019	-3.3 (-6.9, -2.1)			0.2 (-2.6, 3.1)
Native female high education	2004–2014	0.1 (-1.5, 3.2)	2014–2019	-9.5 (-14.5, -6.4)			-3.2 (-4.3, -1.8)

APC: Annual percentage change; AAPC: Average annual percent change.

Numbers in parentheses show 95% confidence intervals.

Table A4. Temporal changes in age-standardized rates of anxiety disorders across sociodemographic strata defined by nativity, sex, and education, 2004-2019.

Stratum	Period 1	APC 1	Period 2	APC 2	Period 3	APC 3	AAPC (2004–2019)
First-generation immigrant males with low education	2004–2015	-3.5 (-4.9, 7.8)	2015–2019	-10.4 (-16.8, -5.6)			-5.4 (-6.6, -2.9)
First-generation immigrant males with medium education	2004–2014	-2.2 (-3.9, 10.5)	2014–2019	-7.2 (-13.4, -3.9)			-3.9 (-5.2, -1.3)
First-generation immigrant males with high education	2004–2019	-4.1 (-5.1, -2.5)					-4.1 (-5.1, -2.5)
First-generation immigrant females with low education	2004–2007	9.6 (0.1, 22.4)	2007–2015	-3.9 (-5.8, -1.8)	2015–2019	-12.3 (-18.1, -9.2)	-3.7 (-5.2, -1.7)
First-generation immigrant females with medium education	2004–2014	-0.8 (-2.1, 7.2)	2014–2019	-6.9 (-12.4, -3.7)			-2.9 (-3.9, -0.8)
First-generation immigrant females with high education	2004–2014	-0.6 (-1.6, 9.6)	2014–2019	-4.9 (-8.8, -2.6)			-2.0 (-2.8, 0.2)
Second-generation immigrant males with low education	2004–2014	3.4 (1.3, 9.7)	2014–2019	-7.9 (-17.0, -2.8)			-0.5 (-2.4, 1.9)
Second-generation immigrant males with medium education	2004–2019	-0.2 (-1.2, 1.0)					-0.2 (-1.2, 1.0)
Second-generation immigrant males with high education	2004–2019	1.1 (-0.4, 3.2)					1.1 (-0.4, 3.2)
Second-generation immigrant females with low education	2004–2007	19.1 (1.7, 41.3)	2007–2019	-1.7 (-6.4, -0.3)			2.1 (-0.9, 5.3)
Second-generation immigrant females with medium education	2004–2019	1.2 (-0.5, 3.3)					1.2 (-0.5, 3.3)
Second-generation immigrant females with high education	2004–2016	4.3 (2.3, 28.9)	2016–2019	-2.9 (-9.7, 3.5)			2.8 (1.6, 7.5)
Native male low education	2004–2014	2.7 (1.5, 9.4)	2014–2019	-3.5 (-10.4, -0.1)			0.6 (-0.6, 2.5)
Native male medium education	2004–2015	1.1 (0.1, 13.3)	2015–2019	-2.9 (-8.3, 0.2)			0.0 (-0.9, 2.5)
Native male high education	2004–2014	4.5 (3.1, 12.4)	2014–2019	-2.2 (-8.1, 0.8)			2.2 (1.1, 4.7)
Native female low education	2004–2007	10.6 (0.3, 23.8)	2007–2019	-1.2 (-6.5, -0.1)			1.1 (-1.2, 3.1)
Native female medium education	2004–2019	0.7 (-0.5, 2.2)					0.7 (-0.5, 2.2)
Native female high education	2004–2016	3.6 (0.5, 27.8)	2016–2019	-1.7 (-8.3, 4.4)			2.5 (1.5, 7.1)

APC: Annual percentage change; AAPC: Average annual percent change.

Numbers in parentheses show 95% confidence intervals.

Table A5. Temporal changes in age-standardized rates of personality disorders across sociodemographic strata defined by nativity, sex, and education, 2004-2019.

Stratum	Period 1	APC 1	Period 2	APC 2	Period 3	APC 3	AAPC (2004–2019)
First-generation immigrant males with low education	2004–2007	18.7 (2.8, 40.1)	2007–2013	-7.6 (-17.8, -2.4)	2013–2019	-16.6 (-29.0, -9.3)	-6.8 (-9.5, -4.1)
First-generation immigrant males with medium education	2004–2007	15.3 (-2.2, 36.0)	2007–2019	-7.4 (-15.3, -5.4)			-3.2 (-6.4, -0.2)
First-generation immigrant males with high education	2004–2009	13.1 (-1.9, 76.8)	2009–2019	-8.4 (-22.4, -4.8)			-1.7 (-6.9, 6.5)
First-generation immigrant females with low education	2004–2015	-4.2 (-5.9, -1.0)	2015–2019	-19.4 (-28.9, -12.0)			-8.5 (-10.2, -6.7)
First-generation immigrant females with medium education	2004–2019	-6 (-8.4, -3.2)					-6 (-8.4, -3.2)
First-generation immigrant females with high education	2004–2007	44.8 (8.0, 85.1)	2007–2019	-6.4 (-10.6, -3.8)			2.1 (-1.8, 6.9)
Second-generation immigrant males with low education	2004–2019	-1.5 (-4.5, 1.7)					-1.5 (-4.5, 1.7)
Second-generation immigrant males with medium education	2004–2019	-5.5 (-8.1, -2.4)					-5.5 (-8.1, -2.4)
Second-generation immigrant males with high education	2004–2008	17.5 (0.3, 64.1)	2008–2019	-3.3 (-10.3, -1.0)			1.9 (-2.2, 7.6)
Second-generation immigrant females with low education	2004–2010	9.2 (-0.3, 48.7)	2010–2019	-8.9 (-22.4, -4.8)			-2 (-6.6, 3.5)
Second-generation immigrant females with medium education	2004–2007	15.8 (-1.0, 36.9)	2007–2019	-2.6 (-7.3, -1.1)			0.8 (-2.3, 4.1)
Second-generation immigrant females with high education	2004–2008	26.3 (7.6, 59.9)	2008–2019	-3 (-8.7, 0.1)			4.1 (0.8, 8.0)
Native male low education	2004–2014	0.6 (-1.2, 6.7)	2014–2019	-9.2 (-19.0, -4.2)			-2.8 (-4.6, -0.6)
Native male medium education	2004–2019	-3.6 (-4.9, -2.1)					-3.6 (-4.9, -2.1)
Native male high education	2004–2019	-2.2 (-3.6, -0.4)					-2.2 (-3.6, -0.4)
Native female low education	2004–2007	14.6 (2.9, 25.3)	2007–2019	-3.1 (-5.3, -1.9)			0.2 (-1.5, 1.8)
Native female medium education	2004–2007	17.2 (2.0, 36.0)	2007–2019	-1.5 (-4.3, -0.4)			2 (-0.4, 4.8)
Native female high education	2004–2016	0.7 (-0.2, 4.9)	2016–2019	-8.2 (-12.8, -2.1)			-1.2 (-2, 0.7)

APC: Annual percentage change; AAPC: Average annual percent change.

Numbers in parentheses show 95% confidence intervals.

Table A6. Temporal changes in age-standardized rates of autism spectrum disorders and other pervasive developmental disorders across sociodemographic strata defined by nativity, sex, and education, 2004-2019.

Stratum	Period 1	APC 1	Period 2	APC 2	Period 3	APC 3	AAPC (2004–2019)
First-generation immigrant males with low education	2004–2014	9.0 (4.1, 25.6)	2014–2019	-15.9 (-32.8, -5.5)			0.0 (-4.1, 6.2)
First-generation immigrant males with medium education	2004–2010	27.4 (14.5, 72.0)	2010–2019	-2 (-9.4, 2.2)			8.8 (5.2, 15.8)
First-generation immigrant males with high education	2004–2008	64.2 (13, 274.7)	2008–2015	9.3 (-2.7, 30.2)	2015–2019	-10.6 (-28.9, 1.6)	15.5 (8.5, 33.0)
First-generation immigrant females with low education	2004–2010	33.3 (10.4, 279.6)	2010–2019	-2.6 (-22.4, 5.0)			10.4 (2.3, 31.5)
First-generation immigrant females with medium education	2004–2011	32.2 (21.9, 55.1)	2011–2019	-6 (-12.4, -1.4)			10.2 (7.0, 15.6)
First-generation immigrant females with high education	2004–2010	45.8 (21.8, 163.4)	2010–2019	5.5 (-3.1, 10.9)			20.1 (13.7, 33.3)
Second-generation immigrant males with low education	2004–2012	16.7 (9.2, 70.7)	2012–2019	-3.4 (-16.9, 2.8)			6.9 (2.7, 16.0)
Second-generation immigrant males with medium education	2004–2009	45.0 (21.7, 121.4)	2009–2019	0.9 (-5.1, 5.0)			13.9 (8.8, 21.3)
Second-generation immigrant males with high education	2004–2012	24.8 (15.5, 80)	2012–2019	-3.4 (-19.2, 4.3)			10.7 (5.8, 22.3)
Second-generation immigrant females with low education	2004–2014	15.3 (6.4, 110.6)	2014–2019	0.2 (-22.0, 13.9)			10.1 (5.8, 23.2)
Second-generation immigrant females with medium education	2004–2008	54.2 (32.3, 114.0)	2008–2013	12.8 (-0.6, 24.2)	2013–2019	1.1 (-9.0, 8.8)	17.4 (14.2, 24.2)
Second-generation immigrant females with high education	2004–2009	72.6 (48.7, 138.8)	2009–2019	5 (2.3, 7.8)			23.9 (20.4, 31.7)
Native male low education	2004–2011	15.0 (9.4, 35.3)	2011–2019	0.7 (-5.0, 3.7)			7.1 (4.9, 11.2)
Native male medium education	2004–2007	54.3 (34.7, 80.1)	2007–2013	9.4 (5.7, 14.8)	2013–2019	1.1 (-4.6, 3.4)	13.6 (11.8, 16.9)
Native male high education	2004–2008	50.0 (24.1, 104.8)	2008–2019	3.8 (0.3, 6.7)			14.5 (10.9, 19.9)
Native female low education	2004–2012	25.1 (20.1, 33.5)	2012–2019	-0.8 (-5.1, 2.6)			12.3 (10.4, 14.9)
Native female medium education	2004–2007	65.7 (41.0, 96.8)	2007–2012	17.8 (9.3, 24.8)	2012–2019	4.6 (-0.2, 6.9)	19.3 (17.0, 23.1)
Native female high education	2004–2012	25.4 (18.5, 57.1)	2012–2019	5.7 (-1.0, 10)			15.8 (13.0, 23.5)

APC: Annual percentage change; AAPC: Average annual percent change.

Numbers in parentheses show 95% confidence intervals.

Table A7. Temporal changes in age-standardized rates of attention-deficit hyperactivity disorder and conduct disorder across sociodemographic strata defined by nativity, sex, and education, 2004-2019.

Stratum	Period 1	APC 1	Period 2	APC 2	Period 3	APC 3	AAPC (2004–2019)
First-generation immigrant males with low education	2004–2008	56.5 (38.7, 97.6)	2008–2014	3.2 (-0.9, 10.3)	2014–2019	-10.7 (-17.3, -7.0)	9.9 (7.5, 14.1)
First-generation immigrant males with medium education	2004–2008	61.0 (38.6, 138.3)	2008–2014	7.7 (3.4, 15.1)	2014–2019	-5.0 (-12.1, -1.5)	15.0 (11.6, 22.4)
First-generation immigrant males with high education	2004–2011	30.5 (16.9, 84.2)	2011–2019	0.8 (-5.8, 5.3)			13.7 (9.4, 23.9)
First-generation immigrant females with low education	2004–2007	95.2 (53.8, 143.2)	2007–2013	5.8 (1.4, 12.7)	2013–2019	-5.1 (-11.7, -2.2)	14.5 (10.9, 19.3)
First-generation immigrant females with medium education	2004–2007	95.3 (61.0, 123.0)	2007–2015	11.8 (9.5, 14.7)	2015–2019	-5.2 (-9.6, -1.7)	19.6 (17.2, 22.8)
First-generation immigrant females with high education	2004–2007	107.8 (38.4, 273.4)	2007–2012	28.7 (-4.2, 44.4)	2012–2019	2.0 (-4.7, 9.9)	27.1 (19.7, 42.4)
Second-generation immigrant males with low education	2004–2008	65.2 (45.3, 99.3)	2008–2015	6.0 (3.1, 11.4)	2015–2019	-8.1 (-15.2, -3.3)	14.8 (12.3, 18.5)
Second-generation immigrant males with medium education	2004–2007	94.7 (58.8, 162.2)	2007–2014	11.2 (7.4, 17.5)	2014–2019	-2.5 (-9.4, 1.3)	19.1 (16.3, 26.2)
Second-generation immigrant males with high education	2004–2011	48.5 (38.2, 70.4)	2011–2019	5.2 (1.7, 8.3)			23.5 (20.7, 29.3)
Second-generation immigrant females with low education	2004–2009	55.7 (42.2, 76.6)	2009–2016	3.5 (1.6, 8.2)	2016–2019	-6.9 (-11.9, -1.3)	16.1 (13.8, 19.7)
Second-generation immigrant females with medium education	2004–2008	82.4 (60.2, 149.5)	2008–2012	17.8 (9.2, 29.2)	2012–2019	3.9 (0.5, 5.7)	24.9 (21.9, 31.4)
Second-generation immigrant females with high education	2004–2010	58.6 (36.6, 144.7)	2010–2019	6.3 (1.2, 10.7)			24.8 (19.9, 39.0)
Native male low education	2004–2008	70.8 (51.4, 97.4)	2008–2014	8.1 (5.2, 13.5)	2014–2019	-2.6 (-9.3, 0.5)	18.0 (15.8, 22.0)
Native male medium education	2004–2008	75.5 (44.1, 126.2)	2008–2019	4.3 (2.8, 5.8)			19.8 (16.0, 27.2)
Native male high education	2004–2007	90.6 (44.3, 183.5)	2007–2012	22.9 (6.2, 33.3)	2012–2019	4.0 (-0.8, 7.1)	24.1 (20.2, 34.1)
Native female low education	2004–2009	67.4 (45.4, 118.7)	2009–2019	2.5 (-0.3, 5.2)			20.7 (17.1, 28.1)
Native female medium education	2004–2009	67.3 (49.6, 84.5)	2009–2019	6.1 (4.7, 7.6)			23.5 (21.1, 27.5)
Native female high education	2004–2007	99.9 (54.4, 168.3)	2007–2011	27.0 (7.4, 36.2)	2011–2019	6.6 (3.8, 8.6)	26.7 (23.1, 33.9)

APC: Annual percentage change; AAPC: Average annual percent change.

Numbers in parentheses show 95% confidence intervals.

Table A8. Cumulative age-standardized incidence rates (95% CI) of specific psychiatric disorders per 10,000 person years among people aged 25-46 years during 2004-2019 across sociodemographic strata defined by nativity, sex, and education.

Stratum	F10-F19	F20, F23	F32-F33	F40-F41, F43	F60, F63, F68	F84	F90-F91
First-generation immigrant males with low education	41.4 (40.0, 42.8)	10.2 (9.5, 10.9)	39.0 (37.6, 40.3)	74.5 (72.5, 76.4)	7.6 (7.0, 8.2)	2.8 (2.5, 3.2)	9.7 (9.1, 10.4)
First-generation immigrant males with medium education	31.6 (30.8, 32.5)	7.1 (6.7, 7.5)	34.5 (33.6, 35.4)	56.7 (55.6, 57.8)	5.9 (5.5, 6.3)	1.9 (1.7, 2.1)	8.5 (8.1, 8.9)
First-generation immigrant males with high education	12.8 (12.4, 13.3)	3.1 (2.9, 3.3)	21.4 (20.8, 22.0)	34.5 (33.8, 35.3)	2.4 (2.2, 2.6)	1.1 (0.9, 1.2)	4.4 (4.2, 4.7)
First-generation immigrant females with low education	16.0 (15.1, 16.9)	7.3 (6.7, 7.9)	53.6 (51.9, 55.2)	95.2 (92.9, 97.4)	7.3 (6.7, 7.9)	1.7 (1.4, 2.0)	5.8 (5.3, 6.3)
First-generation immigrant females with medium education	15.7 (15.0, 16.3)	4.7 (4.3, 5.0)	52.2 (51.1, 53.4)	82.9 (81.4, 84.3)	7.2 (6.8, 7.6)	1.7 (1.5, 1.9)	7.1 (6.7, 7.6)
First-generation immigrant females with high education	7.6 (7.2, 7.9)	2.9 (2.7, 3.1)	35.7 (34.9, 36.4)	58.1 (57.1, 59.0)	3.7 (3.4, 3.9)	1.1 (1.0, 1.2)	4.4 (4.2, 4.7)
Second-generation immigrant males with low education	94.0 (90.4, 97.6)	16.6 (15.1, 18.0)	61.4 (58.6, 64.2)	101.1 (97.4, 104.7)	21.7 (20.0, 23.3)	13.6 (12.3, 14.9)	49.4 (46.9, 51.9)
Second-generation immigrant males with medium education	43.1 (41.9, 44.2)	7.1 (6.7, 7.6)	39.0 (37.9, 40.1)	57.2 (55.9, 58.5)	8.7 (8.2, 9.2)	6.0 (5.5, 6.4)	21.1 (20.3, 21.9)
Second-generation immigrant males with high education	17.2 (16.4, 18.1)	3.4 (3.1, 3.8)	28.2 (27.1, 29.2)	39.0 (37.7, 40.2)	4.5 (4.1, 5.0)	4.0 (3.6, 4.4)	9.9 (9.3, 10.6)
Second-generation immigrant females with low education	70.7 (66.7, 74.8)	12.6 (10.9, 14.2)	103.5 (98.6, 108.4)	169.4 (163.0, 175.9)	35.2 (32.5, 37.9)	14.1 (12.4, 15.8)	55.0 (51.6, 58.4)
Second-generation immigrant females with medium education	27.9 (26.9, 28.9)	4.6 (4.2, 5.0)	63.6 (62.0, 65.2)	97.9 (95.9, 99.9)	15.3 (14.6, 16.1)	5.2 (4.7, 5.6)	22.1 (21.2, 23.0)
Second-generation immigrant females with high education	12.0 (11.4, 12.6)	2.7 (2.4, 3.0)	47.2 (45.9, 48.4)	74.0 (72.4, 75.6)	7.9 (7.4, 8.4)	3.0 (2.7, 3.3)	11.2 (10.6, 11.7)
Native male low education	67.0 (65.5, 68.4)	9.9 (9.3, 10.4)	55.0 (53.7, 56.3)	86.9 (85.2, 88.6)	14.6 (13.9, 15.3)	13.7 (13.0, 14.3)	44.4 (43.2, 45.6)
Native male medium education	26.4 (26.1, 26.8)	3.7 (3.5, 3.8)	30.2 (29.8, 30.6)	43.1 (42.6, 43.5)	5.2 (5.1, 5.4)	4.5 (4.4, 4.7)	15.0 (14.7, 15.3)
Native male high education	11.4 (11.1, 11.7)	2.2 (2.1, 2.3)	21.8 (21.4, 22.2)	30.7 (30.3, 31.2)	2.7 (2.6, 2.9)	3.2 (3.1, 3.3)	6.7 (6.5, 6.9)
Native female low education	55.7 (53.8, 57.5)	9.2 (8.5, 9.9)	93.0 (90.6, 95.4)	154.4 (151.2, 157.6)	31.0 (29.7, 32.3)	15.0 (14.1, 15.9)	53.5 (51.7, 55.2)
Native female medium education	19.1 (18.7, 19.4)	2.8 (2.6, 2.9)	49.8 (49.2, 50.4)	75.4 (74.6, 76.1)	10.4 (10.1, 10.6)	4.2 (4.0, 4.4)	17.1 (16.8, 17.5)
Native female high education	8.0 (7.8, 8.2)	1.6 (1.5, 1.7)	35.4 (35.0, 35.8)	54.8 (54.3, 55.4)	4.8 (4.7, 5.0)	2.0 (1.9, 2.1)	7.3 (7.1, 7.5)

Table A9. Average annual percent change in age-standardized rates of psychiatric disorders among people aged 25-46 years across sociodemographic strata defined by nativity, sex, and education, 2004-2019.

Stratum	F10–F19	F20, F23	F32–F33	F40–F41, F43	F60, F63, F68	F84	F90-F91
First-generation immigrant males with low education	-2.0 (-3.1, -0.7)	-5.7 (-8.0, -4.0)	-6.2 (-8.6, -4.2)	-3.3 (-5.2, -1.7)	-8.2 (-10.5, -6.2)	0.3 (-3.4, 5.4)	8.4 (6.6, 11.1)
First-generation immigrant males with medium education	0.0 (-1.4, 1.2)	-3.6 (-5.2, -1.8)	-4.2 (-6.0, -3.0)	-3.2 (-4.5, -2.0)	-4.9 (-6.9, -3.0)	5.8 (2.5, 11.3)	13.4 (11.4, 17.8)
First-generation immigrant males with high education	-0.3 (-1.5, 1.2)	-5.8 (-8.6, -3.0)	-4.2 (-6.4, -2.5)	-2.8 (-3.9, -1.5)	-3.3 (-7.6, 0.6)	9.4 (4.5, 24.5)	14.2 (11.5, 21.2)
First-generation immigrant females with low education	-3.2 (-4.8, -1.7)	-7.3 (-10.2, -4.6)	-7.7 (-9.7, -6.3)	-3.7 (-4.9, -2.7)	-9.7 (-12.0, -7.0)	7.8 (-1.6, 32.0)	11.5 (7.6, 17.9)
First-generation immigrant females with medium education	-0.1 (-0.8, 0.7)	-6.5 (-8.7, -4.6)	-4.0 (-5.8, -2.7)	-0.8 (-2.5, 0.4)	-3.4 (-5.9, -1.0)	6.6 (0.7, 16.6)	18.0 (15.1, 22.6)
First-generation immigrant females with high education	-0.1 (-1.6, 1.7)	-5.2 (-6.7, -3.7)	-3.3 (-5.5, -1.3)	0.0 (-1.4, 1.1)	0.5 (-4.9, 5.7)	17.1 (12.0, 28.1)	25.4 (21.5, 31.6)
Second-generation immigrant males with low education	-0.1 (-1.5, 0.9)	-0.9 (-3.4, 1.6)	1.7 (-0.3, 3.6)	-0.1 (-2.7, 2.6)	-1.6 (-4.1, 0.8)	7.0 (3.8, 12.4)	13.8 (11.3, 17.3)
Second-generation immigrant males with medium education	0.7 (-0.7, 2.2)	-1.4 (-3.8, 0.9)	1.3 (-0.8, 3.0)	1.0 (-0.2, 2.9)	-2.2 (-4.8, 0.5)	11.9 (7.4, 20.9)	18.2 (15.7, 23.9)
Second-generation immigrant males with high education	2.6 (1.2, 4.5)	-0.9 (-4.0, 1.6)	0.7 (-2.0, 4.6)	2.4 (1.1, 4.3)	-0.6 (-3.0, 1.8)	10.0 (5.9, 17.3)	22.1 (19.7, 26.2)
Second-generation immigrant females with low education	0.2 (-1.4, 1.7)	-5.3 (-9.8, -1.6)	-0.4 (-2.2, 1.5)	1.4 (-0.2, 2.8)	-4.0 (-6.6, -1.6)	10.2 (6.8, 19.9)	17.4 (15.7, 20.1)
Second-generation immigrant females with medium education	1.8 (0.0, 3.7)	-4.8 (-7.0, -2.9)	1.1 (-1.0, 2.5)	2.3 (0.7, 3.9)	2.6 (0.6, 4.6)	16.1 (12.0, 24.7)	24.0 (21.0, 28.6)
Second-generation immigrant females with high education	2.0 (-0.4, 4.7)	-3.9 (-8.1, 0.0)	-0.1 (-1.9, 1.6)	4.0 (2.3, 6.1)	1.2 (-0.8, 3.5)	21.3 (19.0, 27.0)	22.1 (18.2, 35.2)
Native male low education	1.5 (0.3, 2.4)	-0.8 (-2.7, 0.8)	2.0 (0.6, 3.5)	1.6 (0.1, 3.0)	-2.0 (-4.4, -0.2)	8.6 (5.9, 12.7)	17.6 (15.7, 20.7)
Native male medium education	1.0 (0.6, 1.4)	-1.8 (-2.9, -0.7)	1.3 (0.1, 2.4)	1.3 (0.3, 2.7)	-0.4 (-2.8, 1.2)	11.9 (10.7, 13.8)	21.8 (19.8, 24.8)
Native male high education	2.9 (1.6, 5.4)	-3.3 (-5.2, -1.6)	0.3 (-1.9, 3.0)	2.9 (2.1, 4.0)	0.8 (-2.6, 3.5)	11.0 (8.2, 16.2)	24.1 (21.9, 29.1)
Native female low education	1.6 (0.8, 2.3)	-3.0 (-4.8, -0.6)	1.0 (-0.1, 2.2)	1.8 (0.0, 3.2)	0.1 (-2.0, 1.5)	11.9 (10.0, 14.7)	20.7 (17.1, 28.6)
Native female medium education	2.3 (1.7, 2.9)	-3.5 (-6.1, -1.6)	0.7 (-0.8, 1.9)	2.7 (1.2, 3.8)	3.1 (2.0, 4.1)	17.9 (15.3, 22.3)	22.1 (20.0, 27.6)
Native female high education	3.1 (1.9, 4.5)	-2.9 (-4.2, -1.8)	-1.8 (-3.3, -0.3)	3.6 (2.7, 4.8)	2.6 (1.1, 3.8)	16.6 (14.2, 23.2)	24.5 (22.0, 28.8)

Table A10. Cumulative age-standardized incidence rates (95% CI) of specific psychiatric disorders per 10,000 person-years among people aged 25-46 years in 2004 (closed cohort) across sociodemographic strata defined by nativity, sex, and education, 2004-2019.

	F10-F19	F20, F23	F32-F33	F40-F41, F43	F60, F63, F68	F84	F90-F91
First-generation immigrant males with low education	39.1 (37.2, 41.0)	10.1 (9.1, 11.0)	45.2 (43.2, 47.1)	71.2 (68.6, 73.7)	9.2 (8.2, 10.1)	2.7 (2.2, 3.2)	9.4 (8.5, 10.3)
First-generation immigrant males with medium education	28.8 (27.9, 29.8)	6.3 (5.8, 6.7)	39.1 (38.0, 40.2)	57.5 (56.2, 58.9)	6.0 (5.6, 6.5)	1.6 (1.4, 1.8)	6.9 (6.4, 7.4)
First-generation immigrant males with high education	12.8 (12.1, 13.4)	3.0 (2.7, 3.3)	27.6 (26.6, 28.5)	38.3 (37.2, 39.5)	3.2 (2.9, 3.5)	1.2 (1.0, 1.4)	3.7 (3.4, 4.1)
First-generation immigrant females with low education	17.9 (16.7 (19.1)	7.5 (6.7, 8.3)	68.2 (65.8, 70.6)	99.5 (96.5, 102.4)	8.0 (7.2, 8.8)	1.4 (1.1, 1.8)	5.7 (5.0, 6.4)
First-generation immigrant females with medium education	16.3 (15.6, 17.0)	4.9 (4.5, 5.3)	58.4 (56.9, 59.8)	84.0 (82.3, 85.7)	6.8 (6.3, 7.3)	1.5 (1.3, 1.7)	6.1 (5.6, 6.5)
First-generation immigrant females with high education	8.3 (7.8, 8.8)	2.9 (2.6, 3.2)	41.6 (40.5, 42.7)	62.7 (61.4, 64.1)	3.8 (3.5, 4.1)	1.1 (1.0, 1.3)	3.8 (3.5, 4.2)
Second-generation immigrant males with low education	70.9 (67.5, 74.3)	11.6 (10.3, 12.9)	46.3 (43.7, 48.9)	75.6 (72.1, 79.0)	15.8 (14.3, 17.4)	7.3 (6.3, 8.3)	27.5 (25.5, 29.5)
Second-generation immigrant males with medium education	37.1 (36.0, 38.3)	5.6 (5.2, 6.0)	32.4 (31.4, 33.4)	46.4 (45.2, 47.7)	6.9 (6.4, 7.4)	3.5 (3.2, 3.9)	13.7 (13.0, 14.3)
Second-generation immigrant males with high education	16.4 (15.5, 17.4)	2.5 (2.2, 2.9)	23.7 (22.6, 24.8)	32.8 (31.5, 34.1)	3.6 (3.2, 4.0)	2.7 (2.3, 3.0)	5.9 (5.3, 6.4)
Second-generation immigrant females with low education	59.3 (55.4, 63.1)	11.3 (9.7, 13.0)	76.5 (72.1, 80.9)	127.2 (121.4, 133.1)	23.2 (20.8, 25.6)	7.1 (5.9, 8.4)	29.7 (27.0, 32.3)
Second-generation immigrant females with medium education	24.3 (23.3, 25.3)	4.1 (3.7, 4.5)	50.3 (48.9, 51.8)	76.7 (74.9, 78.5)	9.9 (9.3, 10.5)	2.7 (2.3, 3.0)	12.3 (11.6, 13.0)
Second-generation immigrant females with high education	11.9 (11.2, 12.6)	2.4 (2.1, 2.7)	39.8 (38.5, 41.0)	61.9 (60.3, 63.5)	5.7 (5.3, 6.2)	2.3 (2.0, 2.6)	7.4 (6.9, 8.0)
Native male low education	47.6 (46.4, 48.9)	7.0 (6.6, 7.5)	37.2 (36.1, 38.3)	57.8 (56.4, 59.1)	9.4 (8.9, 9.9)	6.1 (5.6, 6.5)	20.6 (19.8, 21.4)
Native male medium education	24.4 (24.0, 24.7)	3.0 (2.8, 3.1)	24.7 (24.4, 25.1)	35.1 (34.7, 35.5)	4.0 (3.8, 4.1)	2.5 (2.4, 2.6)	8.9 (8.6, 9.1)
Native male high education	11.8 (11.5, 12.1)	1.9 (1.8, 2.0)	19.2 (18.8, 19.6)	27.1 (26.6, 27.5)	2.3 (2.2, 2.4)	2.2 (2.1, 2.4)	4.4 (4.2, 4.6)
Native female low education	41.1 (39.5, 42.6)	7.6 (7.0, 8.3)	64.8 (62.9, 66.8)	105.4 (102.9, 108.0)	18.2 (17.1, 19.3)	6.1 (5.5, 6.7)	24.6 (23.4, 25.7)
Native female medium education	16.6 (16.3, 17.0)	2.6 (2.5, 2.7)	38.6 (38.0, 39.1)	58.9 (58.3, 59.6)	6.5 (6.2, 6.7)	2.0 (1.9, 2.1)	8.8 (8.5, 9.0)
Native female high education	7.9 (7.7, 8.1)	1.6 (1.5, 1.7)	30.1 (29.7, 30.6)	47.5 (46.9, 48.0)	3.2 (3.1, 3.4)	1.3 (1.3, 1.4)	4.8 (4.6, 4.9)

Table A11. Average annual percent change in age-standardized rates of psychiatric disorders among people aged 25-46 years in 2004 (closed cohort) across sociodemographic strata defined by nativity, sex, and education, 2004-2019.

Stratum	F10–F19	F20, F23	F32–F33	F40–F41, F43	F60, F63, F68	F84	F90-F91
First-generation immigrant males with low education	-2.5 (-4.7, -0.7)	-10.0 (-14.4, -6.4)	-5.0 (-7.3, -3.1)	-3.8 (-7.2, -1.1)	-3.6 (-10.5, -0.1)	-3.8 (-12.3, 4.1)	17.6 (12.8, 24.0)
First-generation immigrant males with medium education	-1.4 (-3.5, -0.2)	-8.3 (-16.6, -5.3)	-3.3 (-9.0, -0.9)	-2.3 (-4.3, -0.6)	-9.2 (-15.8, -2.0)	1.2 (-7.4, 9.3)	17.1 (9.7, 26.0)
First-generation immigrant males with high education	-2.9 (-6.1, 1.0)	-12.3 (-17.2, -8.1)	-5.9 (-8.8, -3.2)	-3.2 (-5.9, -0.6)	5.4 (-4.8, 10.6)	5.9 (-3.3, 21.9)	14.6 (8.3, 21.4)
First-generation immigrant females with low education	2.2 (-5.2, 4.6)	-9.2 (-13.2, -5.8)	-7.3 (-11.5, -4.1)	-6.4 (-9.6, -3.8)	-12.3 (-18.5, -8.7)	-31.8 (-39.7, -21.4)	11.4 (5.3, 18.8)
First-generation immigrant females with medium education	0.9 (-3.3, 4.0)	-8.8 (-13.7, -4.7)	-2.7 (-5.7, -1.4)	0.7 (-2.9, 2.4)	-10.0 (-18.0, 0.5)	9.4 (-0.8, 20.2)	21.5 (12.8, 30.1)
First-generation immigrant females with high education	0.2 (-2.0, 2.7)	-5.5 (-9.3, -2.7)	-1.9 (-8.5, 1.8)	0.2 (-1.1, 1.5)	5.0 (-3.4, 9.2)	20 (8.6, 37.3)	29.4 (17.9, 45.1)
Second-generation immigrant males with low education	-3.1 (-5.3, -1.4)	-8.1 (-10.6, -6.3)	-0.7 (-5.1, 2.9)	-0.2 (-3.1, 1.5)	0.8 (-4.9, 5.3)	0.0 (-10.1, 16.1)	11.0 (5.0, 15.8)
Second-generation immigrant males with medium education	-2.3 (-3.7, -1.0)	-0.3 (-6.4, 3.4)	-2.5 (-3.9, -1.2)	-0.2 (-1.8, 1.3)	-10.8 (-17.2, -1.8)	9.8 (-0.3, 20.7)	18.6 (13.9, 23.5)
Second-generation immigrant males with high education	1.0 (-1.5, 3.7)	-8.0 (-16.4, -1.0)	-3.2 (-8.4, 3.5)	2.4 (-3.1, 6.9)	-7.6 (-16.5, 8.4)	6.0 (0.9, 12.5)	22.6 (15.4, 34.3)
Second-generation immigrant females with low education	0.8 (-6.4, 5.0)	-7.4 (-13.3, -2.8)	0.5 (-4.2, 2.5)	1.6 (-6.3, 4.8)	-11.4 (-21.5, -3.8)	1.6 (-6.6, 11.6)	15.4 (10.2, 23.3)
Second-generation immigrant females with medium education	0.1 (-2.5, 2.4)	-8.4 (-12.4, -5.2)	-1.9 (-5.0, 0.6)	3.4 (1.5, 5.4)	-3.7 (-7.0, -1.0)	8.2 (4.1, 15.0)	19.4 (15.6, 24.7)
Second-generation immigrant females with high education	0.5 (-2.1, 3.4)	-7.4 (-12.9, -2.3)	-1.1 (-5.7, 1.5)	3.7 (1.3, 6.2)	3.4 (-2.5, 6.8)	14.6 (8.4, 23.6)	26.3 (19.3, 37.4)
Native male low education	-2.0 (-3.6, -0.8)	-4.5 (-6.6, -2.8)	0.8 (-4.1, 2.7)	0.5 (-4.3, 2.2)	-6.3 (-11.9, -2.4)	0.2 (-3.6, 2.7)	14.2 (10.5, 17.6)
Native male medium education	-1.2 (-3.0, 0.2)	-5.2 (-8.8, -1.9)	-1.0 (-3.2, 0.6)	0.6 (-1.8, 1.9)	-4.5 (-6.8, -2.5)	10.4 (7.7, 12.3)	18.1 (13.6, 29.7)
Native male high education	3.9 (0.1, 6.9)	-5.8 (-10.9, -2.4)	-2.4 (-5.3, 0.3)	1.3 (-1.8, 5.3)	-2.1 (-4.5, 0.3)	13.4 (5.7, 21.4)	20.9 (14.3, 31.6)
Native female low education	-1.1 (-2.6, 0.1)	1.7 (-5.9, 6.7)	-1.0 (-7.2, 1.4)	-0.4 (-3.0, 0.8)	-3.4 (-7.9, -1.7)	14.3 (10.8, 17.2)	18.0 (13.1, 25.0)
Native female medium education	1.1 (0.2, 1.8)	-7.8 (-11.5, -4.7)	-0.8 (-8.2, 2.0)	0.4 (-1.6, 2.0)	-0.3 (-5.9, 1.8)	18.0 (15.1, 21.2)	22.5 (19.0, 27.6)
Native female high education	1.5 (-0.3, 3.6)	-6.0 (-8.1, -4.0)	-5.2 (-8.1, -2.9)	3.0 (1.1, 4.9)	-1.2 (-5.9, 1.5)	15.2 (9.7, 21.6)	26.1 (21.4, 33.8)
